# Acknowledgement to Reviewers of *Diagnostics* in 2019

**DOI:** 10.3390/diagnostics10010053

**Published:** 2020-01-20

**Authors:** Diagnostics Editorial Office

**Affiliations:** MDPI, St. Alban-Anlage 66, 4052 Basel, Switzerland

The editorial team greatly appreciates the reviewers who have dedicated their considerable time and expertise to the journal’s rigorous editorial process over the past 12 months, regardless of whether the papers are finally published or not. In 2019, a total of 234 papers were published in the journal, with a median time to first decision of 15.5 days and a median time from submission to publication of 34.5 days. The editors would like to express their sincere gratitude to the following reviewers for their generous contribution in 2019:
Achreja, AbhinavMarchant, DavidAhamed, MuneerMarcu, LoredanaAhn, YongMariotti, CaterinaAlbano, DomenicoMarkić, JoškoAlbu, SilviuMarks, Haley L.Alizargar, JavadMarotte, HubertAmato, Dominick J.Marshall, Andrew G.Amiya, EisukeMartelli, EugenioAmza, CatalinMartinelli, GiovanniAnton, DavidMaruyama, HitoshiArmellini, EliaMasala, CarlaAshford, J. WessonMasarone, DanieleAssenza, GiovanniMascitti, MarcoAwan, Shakil AhmedMassaroni, CarloBabichev, SergiiMastropietro, AlfonsoBadea, MihaelaMatalliotakis, MichailBagyinszky, EvaMatikonda, SiddharthBakkour, SoniaMatsuda, ShinjiBalbi, BrunoMauk, Michael G.Banerjee, SudipMayer, ChristopherBanning, AntjeMcCary, Lindsay M.Barbosa, Ana IsabelMcEwan, AlistairBardin, RonMcGregor, Neil R.Baroncini, DamianoMcmorrow, TaraBarra, FabioMcNeill, AlisdairBarzon, LuisaMehta, MeghnaBauckneht, MatteoMehta, SunaliBechsgaard, Rie EriksenMelchor, JuanBelmin, JoëlMendoza-Nuñez, Victor ManuelBelteki, GusztavMercorelli, PaoloBequette, B. WayneMerlino, GiovanniBeretta, Giovanni L.Meshram, NirvedhBhatti, Fazal-ur-RehmanMetz, Stefan W.Bisharat, NaielMihaicuta, StefanBishwajit, GhoseMiller, Jillian VinallBlackburn, JessicaMinaga, KosukeBlandino, GiovanniMishina, MasahiroBoard, MaryMitchell, JohnBoling, Warren W.Mitrakas, LamprosBologna, RonellMoccia, MarcelloBoomer, Jonathan S.Mohamed, Salah A.Borghesi, AndreaMohamed, SharmarkeBorse, VikrantMolenaar, Remco J.Bose, TanimaMöller, SörenBotelho, Monica C.Monni, GiovanniBoublikova, LudmilaMontalto, LuigiBoussios, StergiosMorbelli, SilviaBragazzi, Nicola LuigiMoskot, MartaBueno-Campaña, MercedesMotloch, Lukas JBunaciu, Rodica PMozos, IoanaBurger, Michael CMusch, MarkBurtey, StéphaneMustafine, JamilaCaiati, CarloMusteata, MihaiCamara, Jose SousaMuthupillai, RajaCambridge, GeraldineNaber, KurtCampos Souza, Paulo VitorNabeta, TakeruCañadas-De La Fuente, Guillermo A.Nacul, Luis CCandido Carvalho, KatiaNagaraj, ViniCanetta, ElisabettaNakamura, MasatoCasals Terre, JasminaNakanishi, YoshitakaCascini, Giuseppe LucioNandi, SaikatCasiraghi, ElenaNarayanan, BarathCastren, MaijaNardini, RobertoCatry, BoudewijnNath, ShubhankarCavoretto, PaoloNedoma, JanCederbaum, StephenNemeș, Roxana MariaChakravarty, ShatadruNeogi, UjjwalChalopin, ClaireNeri, FlaviaChang, Chia-ChenNeuhaus, JochenChang, Hsien-ChangNeumann, EwaldChang, Ke-VinNguyen, BinhChaudhari, BimalNguyen, Binh P.Chauhan, BhareshNicolini, AntonelloChaves, RaquelNITTA, NaotakaChen, Ke-ChengNobre, Miguel De AraújoChen, Kuan-FuNohe, AnjaChen, Yu-ChunNoll-Hussong, MichaelCheng, XiNonaka, TaichiroCheng, XiangNouri, AriaChien, Yin-HsiuNovo-Veleiro, IgnacioChinnappan, MahendranOakey, ZackChioncel, OvidiuObreza, AlešChladek, GrzegorzOchi, ShinichiroChoi, HojongOftadeh, ShahinChoi, SangchunO’Harte, FinbarrChoo, Pei LingOlesberg, JonathonChuah, Seng-KeeO’Loughlin, DeclanCiaccio, MarcelloOosterloo, MaykeCiudin, AndreaOpdenakker, GhislainCiuoli, CristinaOrsini, JoeClaros, EdithOstovar, SaeidehCoccheri, SergioOszutowska-Mazurek, DorotaColussi, Gian LucaOtomo, TakanobuComi, CristoforoOtsuki, TakemiConverse, Paul J.Ozen, MehmetCorda, AndreaPadwal, RajCorrochano, SilviaPal Choudhuri, ShreoshiCorsi, CristianaPalazón-Bru, AntonioCosta, PedroPalestra, GiuseppeCoutinho, Maria FranciscaPalmucci, StefanoCox, Jonathan A.G.Pampaloni, FrancescoCrippa, PaoloPan, LuyingCristache, Corina MarilenaPanzuto, FrancescoCuadra, GiancarloPapazafiropoulou, AthanasiaCzekierdowski, ArturPark, Jong KookDabrowski, FilipPark, Jun-BeomDaimi, HouriaPark, MinDal Ben, MatteoParker, ChristinaDaley, PeterParsons, Lauren N.Dang, ShaonongPascual, Carmen Mª GarcíaDaniellou, RichardPatini, RomeoDatta, RupsaPatro-Małysza, JolantaDayan, MichaelPavlidis, EfstathiosDe Lorenzo, Mariana SPavlik, EdwardDe Stefano, RosaPavlik, Edward J.Delfino, InesPavlov, Chavdar SDesai, NiyatiPavlova, ElenaDevarajan, PriyadharshiniPeitsidis, PanagiotisDi Donato, MarziaPelin, MarcoDi Gianfilippo, RiccardoPennington, CatherineDinets, Andrii V.Pereira, FilipeDinic, JelenaPergament, EugeneDionysopoulos, DimitriosPezzuto, AldoDocea, Anca OanaPiciu, DoinaDomenici, GiacomoPimenoff, Ville N.Dörr, StefanPinter, AbrahamDrago, LorenzoPiotrowska, EwaDragomir, MihneaPiskin, SenolDubey, HarishchandraPitocco, DarioDusi, VeronicaPlaza-Díaz, JulioEboigbodin, KevinPokorn, MarkoEcke, ThorstenPoku, Rosemary A.Edmund Kim, EuishinPop, Tudor LucianEkpo, Ernest U.Potaczek, Daniel PEl Hadi, HamzaPouliopoulos, AntoniosElhabian, Shireen Y.Pradotto, Luca GuglielmoEmfietzoglou, DimitrisPuniya, Bhanwar LalEngelhardt, Paul FriedrichPuri, KritiEstanis, NavarroPuttreddy, RakeshEsteban, EmilioQian, XuejunFacciorusso, AntonioQiu, XinFalzone, LucaQu, NingFang, XiaolanQuartu, MarinaFanti, AlessandroQuinlan, JonathanFarber, Harrison W.Rahib, LolaFarina, Nicholas H.Rahman, HafizurFasoula, AngieRahmanuddin, SyedFernández-Ochoa, ÁlvaroRai, VikrantFerrara, PietroRaimondo, DiegoFerraris, ClaudiaRajavenkatanarayanan, AkileshField, MichaelRamadas, AmuthaFilella, XavierRamaraj, PanduranganFilius, AnikaRamić, SnježanaFiorillo, LucaRamirez-Gonzalez, GustavoFitsiori, AikateriniRamsingh, DavinderFleischer, Arthur C.Ranganathan, ShivakumarFolkvardsen, Dorte BekRatajczak, MonikaForest, FabienRawla, PrashanthForget, PatriceRay, SupriyoFountas, KostasRehman, MichaelFranco, LuisReiterer, FlorianFreitas, Leandro OliveiraReychler, HervéFthenakis, GeorgeReynard, OlivierFujigaki, MotoharuRezania, ShahabaldinFukumoto, ManabuRizzolio, FlavioFukushima, HiroshiRoberts, Drucilla J.Furmańczyk, KonradRocca, CarmineGalasso, GiovanniRocca, ElenaGallardo, EnriqueRočka, SauliusGallotta, ValerioRodger, James A.Galvosas, PetrikRodríguez Hermosa, Juan LuisGama, AdelinaRomero Morales, CarlosGanetzky, RebeccaRomine, WilliamGao, Shiqian SherryRoque, AliciaGarcia-Diaz, JuliaRosado, CarlosGarrido, Juan E.Rosenzweig, AllisonGasca-Salas, CarmenRoss, J. KimGasperini, SerenaRoy, DhruvajyotiGerke, OkeRus, GuillermoGermain, ArnaudRussell, David G.Gheorghiu, EugenSaha, ProgyaparamitaGhiran, Ionita C.Sahoo, SubhransuGiacomini, MauroSaijo, YoshifumiGijón-Nogueron, GabrielSalkini, MohamadGiovagnoli, RaffaelaSampaio, PaulaGiraldo, PilarSanchez Bornot, Jose MiguelGjörloff Wingren, AnetteSanthanam, AbiramiGladysheva, Inna P.Sardu, CelestinoGoldoni, MatteoSato, KeisakuGomez, AntonioSavopoulos, ChristosGómez-Urquiza, Jose L.Saxer, FranziskaGonzález-López, José RafaelScaravilli, VittorioGonzález-Vallinas, MargaritaScarpa, MaurizioGorte, BenSchelhaas, SonjaGoyal, RajniSchepisi, GiuseppeGraziani, MaristellaSchiltenwolf, MarcusGrewal, Raji PaulSchirmer, LucasGuerra, FloraSchmidl, DoreenGundapaneni, DineshSchob, StefanGuo, QihaoScholkmann, FelixGupta, VijayalaxmiSchreiber, MichaelHaaparanta-Solin, MerjaScimeca, ManuelHafiane, AnouarSergi, ConsolatoHanai, Jun-ichiSeto, ToshiyukiHanna, Ramy M.Shahi, ShaileshHarmatz, PaulShankar, EswarHartanto, AndreeSharma, IshaHasanian, MostafaSharma, UmakantHasegawa, TakaakiShienfeld, YehudaHashemi, ParastooShimada, YohtaHassan, MohamedShin, Young JooHaszprunar, GerhardShinohara, MitsuruHattori, ToshioShinohara, ShogoHayashi, RyujiSimakov, SrgeyHe, AinaSimoes, JoanaHe, YiSimões, JoãoHendrikse, HarrySimopoulou, MaraHernández-Torres, FranciscoSinclair, AndrewHernandez-Vaquero, DanielSingh, AnupHifumi, ToruSingha, SubhankarHingorani, DinaSivakumar, SushamaHirata, KenjiSivathanu, VivekHołda, Mateusz KrystianSkuja, SandraHolt, KellySladkevicius, PovilasHoly, RichardSlart, Riemer H.J.A.Horai, YoshiroSlavik, RogerHorn, SebastianSłomka, ArturHronek, MiloslavSmani, YounesHu, XiaosuSobrino, Xosé Antón VilaHuang, Tzu-TingSolís García Del Pozo, JuliánHUANG, Wen-ChinSolís-Lemus, José AlonsoHubková, BeátaSonkar, KanchanHughes, KevinSorokin, Maxim I.Hwang, Jae YounSorsa, TimoIacopi, ElisabettaSpeight, NigelIde, KazukiSpínola, HélderIlich-Ernst, JasminkaSpizzo, GilbertImamura, TeruhikoSporea, IoanIngendoh-Tsakmakidis, AlexandraStaderini, EdoardoInglada-Pérez, LucíaStahelin, Robert V.Itri, AngeloStaines, DonaldJain, KartikStanley, Jone A.Jakab, AndrásSteinbichler, Teresa BernadetteJanczar, SzymonStergiou, George S.Janmaimool, PiyapongStiburkova, BlankaJašprová, JanaStoian, DanaJatužis, DaliusStreimikiene, DaliaJáuregui-Vázquez, DanielSu, Mei-TszJeong, Keun-YeongSu, RuiJiménez-Bonilla, Julio FranciscoSuciu, Bogdan AndreiJin, LiSuhail, HamidJing, RanSun, MengJohnen, GeorgSun, ZhonghuaJönsson, DanielSung, Si-IlJørgensen, KasperSurdacki, AndrzejJozwiak, MathieuSweiss, NaderaJuhlin, ChristoferSymonds, Michael E.Jung, ChristianSzubert, SebastianKairemo, KaleviTajiri, NaokiKalfa, NicolasTakebayashi, ShigeoKang, JeonghyunTanaka, NaoroKant, ShivaTang, Kwan HoKanyong, ProsperTappia, ParamjitKarlsson, JoakimTarantino, GiovanniKatrinli, SeymaTarazona-Santabalbina, Francisco JoséKatsanos, Aristeidis H.Teng, Ru-JengKavecan, IvanaTiefenboeck, Thomas M.Kawaguchiya, MitsuyoTodsen, TobiasKayser, KlausTolstikov, Vladimir V.Kazufumi, SuzukiTomatsu, ShunjiKeeley, JaredTombelli, SaraKent, ChristopherTomonaga, ShozoKerman, KaganTong, MingmingKerr, JonathanToriello, FilippoKeutzer, JoanTorres, JorgeKhoo, Bee LuanTorres-Lagares, DanielKhurshid, ZohaibTot, TiborKikuyama, MasatakaTotman, JohnKim, Jee TaekTran, Anh NhatKim, Mee KumTsai, Chia-Hung DylanKim, SeonghoTseng, Kuang-WenKim, SeongjaeTsolakis, Apostolos V.Kimber-Trojnar, ŻanetaTsuchiya, MasahikoKimura, MasahiroTsukamoto, TetsuoKiss, RitaTuchscherr, LorenaKlatt, DieterTzartos, Socrates J.Knowlton, Barbara J.Uberti, DanielaKodali, MaheedharUhl, EberhardKoga, FumitakaUmemura, NaokiKoike, TakahikoUnger, ElizabethKontsevaya, IrinaUpadhyaya, JasbirKoshiyama, MasafumiUrbanelli, LorenaKotyla, Przemyslaw J.Uruha, AkinoriKozieł, EdmundUsai Satta, PaoloKratochwil, ClemensUsmani, ShariqKrishna, Somashekar G.Vaculovičová, MarketaKrupa-Kozak, UrszulaVafiadaki, ElizabethKulkarni, PriyankaVaibhav, KumarKumar, RameshVarga, GergelyKunz, MeikVashist, SandeepKuwahara, TakamichiVashist, Sandeep K.Lacerda, ElianaVeilleux, Louis-NicolasLaganà, Antonio SimoneVelikova, TsvetelinaLai, Wen-FuVenkataraman, RajeshLaiakis, EvageliaVenturella, RobertaLandolfo, ChiaraVerghese, ShilpiLang, DiVidetta, WalterLe, Nguyen Quoc KhanhVidic, JasminaLederer, MartinVilla, ChiaraLee, ChangwonVinall, RuthLee, Cheng-IVitale, Salvatore GiovanniLee, Chii-MingWaldhör, ThomasLee, Huei-JaneWang, DongLee, JieWang, FupengLee, RegentWang, GangLee, TimWang, ShuangyiLee, ZhenghongWang, TaoLeffler, JonatanWang, Tsung-JenLehmann-Che, JacquelineWang, WilliamLepage, CecileWang, YumengLerche, ChristophWegrzyn, GrzegorzLeung, DuncanWei, QingshanLeutenegger, MarcelWilczek, PiotrLi, ChenghuiWillemse, ElineLi, ChunfengWlaź, PiotrLi, LinWu, Sheng-NanLi, Xiang-GuoWu, ShuicaiLidbury, BrettWu, Wei-TingLima, ErnestoWu, Yu-JenLima, IvanWynants, LaureLin, Ching-YuXia, JunfengLin, Hsiang-YuXin, YinLisi, Anthony J.Yamauchi, AkiraLitschauer, BrigitteYan, ShengminLiu, JiaYang, Chul-SuLiu, Wei-MinYang, GuangLiu, YuYang, KunLlull, LauraYaqub, MaqsoodLoffredo, LorenzoYeh, Hong-ZenLombardi, Vincent C.Yeh, Sheng LihLongo, AntonioYong, Kar WeyLópez, DanielYoo, Tag KeunLópez-Pintor Muñoz, Rosa MaríaYoshioka, Ken-ichiLosurdo, GiuseppeYoussof Zadeh, VahabLova, PaolaYu, Yan PingLu, Chia-FengYu, YangLu, Jeng-WeiYves, CombarnousLuce-Fedrow, AlisonZafar, Muhammad SohailLuciani, MirellaZahid, MalihaLuisetto, RobertoZemouri, RyadLuo, ZonghuaZhang, ChiLupien, AndréanneZhang, Daniel XinM’kacher, RadhiaZhang, Haichong K.Machelart, ArnaudZhang, HuanMacín, Stella MarisZhang, JinnanMadala, Hanumantha RaoZhang, YudongMagnus, PerZhang, ZhaoMahshid, SaraZhao, WeiMaia, CláudioZhao, YangMalapelle, UmbertoZheng, MinzhangMandal, SubhamoyZhou, ZhuhuangMandato, ClaudiaZiegler, MagnusMango, LucioZiolkowski, RobertManis, GeorgeZou, LiangManzo, CiroZou, WeiMarateb, Hamid Reza


